# Prekallikrein inhibits innate immune signaling in the lung and impairs host defense during pneumosepsis in mice

**DOI:** 10.1002/path.5354

**Published:** 2019-11-25

**Authors:** Chao Ding, Brendon P Scicluna, Ingrid Stroo, Jack Yang, Joris JTH Roelofs, Onno J de Boer, Alex F de Vos, Peter Nürnberg, Alexey S Revenko, Jeff Crosby, Cornelis van't Veer, Tom van der Poll

**Affiliations:** ^1^ Department of Gastric Surgery, Sun Yat‐sen University Cancer Center, State Key Laboratory of Oncology in South China Collaborative Innovation Center for Cancer Medicine Guangzhou PR China; ^2^ Center of Experimental & Molecular Medicine Amsterdam University Medical Centers, location Academic Medical Center, University of Amsterdam Amsterdam The Netherlands; ^3^ Department of Clinical Epidemiology and Biostatistics, Amsterdam University Medical Centers, location Academic Medical Center University of Amsterdam Amsterdam The Netherlands; ^4^ Department of Pathology, Amsterdam University Medical Centers, location Academic Medical Center University of Amsterdam Amsterdam The Netherlands; ^5^ Cologne Center for Genomics (CCG) University of Cologne Cologne Germany; ^6^ Center for Molecular Medicine Cologne (CMMC) University of Cologne Cologne Germany; ^7^ Drug Discovery Ionis Pharmaceuticals, Inc Carlsbad CA USA; ^8^ Division of Infectious Diseases Amsterdam University Medical Centers, location Academic Medical Center, University of Amsterdam Amsterdam The Netherlands

**Keywords:** prekallikrein, pneumonia, sepsis, *Klebsiella*, contact system, innate immunity, lungs

## Abstract

Prekallikrein (PKK, also known as Fletcher factor and encoded by the gene *KLKB1* in humans) is a component of the contact system. Activation of the contact system has been implicated in lethality in fulminant sepsis models. Pneumonia is the most frequent cause of sepsis. We sought to determine the role of PKK in host defense during pneumosepsis. To this end, mice were infected with the common human pathogen *Klebsiella pneumoniae* via the airways, causing an initially localized infection of the lungs with subsequent bacterial dissemination and sepsis. Mice were treated with a selective PKK‐directed antisense oligonucleotide (ASO) or a scrambled control ASO for 3 weeks prior to infection. Host response readouts were determined at 12 or 36 h post‐infection, including genome‐wide messenger RNA profiling of lungs, or mice were followed for survival. PKK ASO treatment inhibited constitutive hepatic *Klkb1* mRNA expression by >80% and almost completely abolished plasma PKK activity. *Klkb1* mRNA could not be detected in lungs. Pneumonia was associated with a progressive decline in PKK expression in mice treated with control ASO. PKK ASO administration was associated with a delayed mortality, reduced bacterial burdens, and diminished distant organ injury. While PKK depletion did not influence lung pathology or neutrophil recruitment, it was associated with an upregulation of multiple innate immune signaling pathways in the lungs already prior to infection. Activation of the contact system could not be detected, either during infection *in vivo* or at the surface of *Klebsiella in vitro*. These data suggest that circulating PKK confines pro‐inflammatory signaling in the lung by a mechanism that does not involve contact system activation, which in the case of respiratory tract infection may impede early protective innate immunity. © 2019 Authors. *Journal of Pathology* published by John Wiley & Sons Ltd on behalf of Pathological Society of Great Britain and Ireland.

## Introduction

Sepsis is a life‐threatening syndrome featured by an unbalanced host response to infection 1, 2. In spite of substantial advancement in the knowledge of pathophysiological mechanisms involved, sepsis still causes a great health burden and mortality remains high 3, 4. Pneumonia is responsible for an inordinate disease burden worldwide 5 and represents the most common cause of sepsis 3, 4. *Klebsiella pneumoniae* is one of the most frequent Gram‐negative causative agents of pneumonia and sepsis 6, 7.

The host response to bacterial infection involves activation of the contact system 8, 9. The contact system consists of three zymogen factors, Factor (F) XII, FXI, and prekallikrein (PKK), and one cofactor, high‐molecular‐weight kininogen (HK). PKK, once activated by FXIIa to kallikrein, exerts its serine protease activity by cleaving its substrate HK to produce the potent inflammatory mediator bradykinin, which is often referred to as the kallikrein–kinin system. Plasma kallikrein also facilitates the activation of FXII, which initiates the intrinsic pathway of coagulation 8, 9. The contact system may play a dual role in the host response to bacterial infection. Activation of the contact system can occur at the surface of bacterial pathogens and inhibition of the contact system after intraperitoneal infection with *Streptococcus pyogenes* in mice resulted in enhanced dissemination to the spleen 10, 11. In contrast, during acute overwhelming infections, contact system activation may contribute to mortality, at least in part by inducing septic shock 12, 13. Knowledge on how the contact system influences the host response during a gradually progressing bacterial infection, from a local infectious source to systemic dissemination, is limited.

We recently reported that FXII‐deficient mice had lower bacterial burdens and an improved survival in a model that uses a low infectious dose of *K. pneumoniae* administered via the airways, initially resulting in a localized infection contained within the lungs and subsequently in sepsis with distant organ injury 14. Remarkably, in the same model of pneumonia‐derived sepsis, more downstream interventions in the contact system targeting kininogen or bradykinin did not modify the host response, suggesting that components of the contact system may influence innate immunity independent of their established role in the kallikrein–kinin system 15, 16. The present study aimed to determine the role of PKK in the host response to Gram‐negative sepsis caused by pneumonia.

## Materials and methods

### Animals

Male C57Bl/6J mice were purchased from Charles River Inc (Maastricht, The Netherlands) and used at 7–8 weeks of age. The Institutional Animal Care and Use Committee approved all experiments.

### Oligonucleotides

Antisense oligonucleotides (ASOs) were synthesized using an Applied Biosystems 380B automated DNA synthesizer (Applied Biosystems, Waltham, MA, USA) and purified as described previously 17. *PKK* ASO 18 and a non‐specific, scrambled control (Ctrl) ASO were administered subcutaneously, twice weekly, at a dose of 40 mg/kg per week for 3 weeks prior to infection. The ASO dose and treatment regimen were established according to previous mouse studies 18, 19.

### Bacterial cultures

*K. pneumoniae* serotype 2 (43816; ATCC, Manassas, VA, USA) was cultured in Tryptic soy broth (TSB) medium at 37 °C, and log‐phase bacteria were collected and washed for experiments. The curli‐expressing *Escherichia coli* K12 strain (Ymel) and its mutant strain (Ymel‐1) were cultured as described previously 20.

### Experimental design

Pneumonia was induced 4 days after the final treatment with PKK or Ctrl ASO by intranasal inoculation with *K. pneumoniae* [∼7000 colony‐forming units (CFU) in 50 μl of isotonic saline] as described previously 21, 22, 23. Mice were euthanized 12 or 36 h after induction of pneumonia (*n* = 8 per group at each time point) or observed for 10 days (*n* = 20 per group). During the latter experiment, clinical signs were scored as described previously 21. At predefined endpoints, blood was collected in sodium citrate prefilled syringes (volume ratio 4:1); organs were harvested and homogenized; and bacteria quantified as described previously [21–23]. In some experiments, bronchoalveolar lavage fluid (BALF) was collected through flushing the lungs via the trachea three times with 300 μl of 0.2 mm EDTA/PBS.

### Measurement of PKK (*Klkb1*) mRNA

RNA was isolated using an RNA isolation kit (Nucleospin RNA, Macherey‐Nagel, Düren, Germany) and then stored at −80 °C until used for reverse transcription and polymerase chain reaction (PCR). Total RNA was transcribed to cDNA using oligo dT primer and Moloney murine leukemia virus reverse transcriptase (Promega Benelux, Leiden, The Netherlands). Gene expression levels were quantified with SYBR Green reactions on a LightCycler system (LC480; Roche Applied Science, Mannheim, Germany). *Gapdh* was used as a reference. The primer sequences were as follows: *Klkb1*: AGTACCGGAAGAAGTGCCTG (forward) and GTGAAGAAAAGGCAGTTGGG (reverse); *Gapdh*: CTCATGACCACAGTCCATGC (forward) and CACATTGGGGGTAGGAACAC (reverse). Real‐time PCR data were analyzed using LinRegPCR software (v.2014.4) (available at http://linregpcr.nl).

### Histology

Four‐micrometer tissue sections were stained with hematoxylin and eosin (H&E) and scored as previously described 24 with respect to the following parameters: bronchitis, edema, interstitial inflammation, intra‐alveolar inflammation, pleuritis, endothelialitis, and percentage of the lung surface demonstrating confluent inflammatory infiltrate. Each parameter was graded 0–4, with 0 being ‘absent’ and 4 being ‘severe’; the total pathology score was expressed as the sum of the score for all parameters. Granulocyte staining used FITC‐labeled rat anti‐mouse Ly‐6 mAb (clone 1A8; BD Pharmingen, San Diego, CA, USA; catalog number 551460; 1:20 000). The amount of Ly‐6G positivity was expressed as a percentage of the total surface area. Fibrinogen staining on lung sections was performed as described previously 25 using a biotin‐labeled goat anti‐mouse fibrinogen antibody (Accurate Chemical & Scientific, Westbury, NY, USA; catalog number YNGMFBGBio; 1:500). Slides were scanned using an Olympus dotSlide scanner (Olympus, Tokyo, Japan) to generate TIFF images of the full tissue section. Ly‐6G and fibrinogen positivity was measured using ImageJ; the amount of positivity was expressed as a percentage of the total lung surface area 21, 25.

### Western blotting

For PKK and HK immunoblotting analyses, citrated plasma was fractionated on 4–12.5% polyacrylamide gels under reducing conditions and separated proteins were transferred to polyvinylidene difluoride membrane (Roche Diagnostics, Almere, The Netherlands) followed by immunoblotting with goat anti‐mouse PKK antibody (AF2498; R&D Systems, Minneapolis, MN, USA) or mouse monoclonal (13G11) anti‐human PKK (GTX21006; GeneTex, Irvine, CA, USA). Rabbit polycolonal antibody against human full‐length HK was used for HK immunoblotting (Abnova, Taipei City, Taiwan). Horseradish peroxidase‐linked rabbit IgGs were used as secondary antibodies and blots were imaged using chemiluminescence and an ImageQuant LAS 4000 imager (GE Healthcare Life Sciences, Little Chalfont, UK).

### Assays

Cytokines [tumor necrosis factor (TNF)‐α, interleukin (IL)‐6, IL‐1β] and chemokines (CXCL1, CXCL2) were measured using ELISA (Duoset ELISA kits; R&D Systems, Abingdon, UK). Thrombin–anti‐thrombin complexes (TATc) were measured by ELISA (Affinity Biologicals, Ancaster, Canada). Lactate dehydrogenase (LDH), alanine transaminase (ALT), and aspartate transaminase (AST) were measured using a c702 Roche Diagnostics module (Roche Diagnostics, Almere, The Netherlands). Prothrombin time (PT) and activated partial thromboplastin time (aPTT) were measured using an automated coagulation analyzer (Behring Coagulation System, BCS; Siemens Healthcare Diagnostics, Marburg, Germany) with reagents and protocols from the manufacturer; plasma PKK, FXII, and HK activities were determined on the same analyzer using human plasma deficient for PKK, FXII, or HK, respectively, from Technoclone (Vienna, Austria) with normal human plasma as a reference.

### Bacterial binding/activation of contact system

Binding and activation of contact system factors by bacteria were determined essentially as described previously 26. In brief, bacteria in buffer C (containing 15 mm HEPES, 135 mm NaCl, 50 μm ZnCl_2_, pH 7.4) were incubated with citrated mouse or human plasma (1:1 v/v) on a roller for 1 h at room temperature. Bacteria (∼4 × 10^10^ CFU) were then pelleted and washed twice with buffer C. Bound proteins on bacterial surfaces were eluted with 0.1 m glycine (pH 2.0) and analyzed by SDS polyacrylamide gel electrophoresis for detection of PKK and HK and derived activation products.

### RNA microarrays and bioinformatics

RNA was isolated from lung and liver homogenate using a Nucleospin RNA isolation kit (Macherey‐Nagel). Only RNA samples with an integrity number (RIN) greater than 6 (Agilent Bioanalyzer; Agilent Technologies, Amstelveen, The Netherlands) were used for microarray analyses. RNA was hybridized to the mouse Clariom S Assay HT chip (Thermo Fisher Scientific, Waltham, MA, USA). Pre‐processing and quality control of the scans were performed by using the oligo method (version 1.44) 27 and probes were annotated using the platform design information for Affymetrix Clariom_S_Mouse_HT available through Bioconductor 28. Array data were background‐corrected using Robust Multi‐array Average (RMA) and quantiles‐normalized. Microarray quality control was performed by means of the array quality metrics method (version 3.36.0) 29. The occurrence of non‐experimental chip effects was evaluated by the surrogate variable analysis method (version 3.28.0) 30 and corrected using the combat method 31. Probes were filtered by means of a 0.5 variance cut‐off using the genefilter method (version 1.62.0) 32, resulting in 14 564 expressed transcripts. Comparison between groups was performed by moderated t‐statistics implemented in the empirical Bayesian linear models method limma (version 3.36.2) 33. Throughout, Benjamini–Hochberg multiple comparison adjusted probabilities (adjusted *p* < 0.05) were used to determine significance. All analyses were performed in the R statistical computing environment (version 3.5.0). Pathway analysis was performed using Ingenuity pathway analysis (Ingenuity Systems, Qiagen Bioinformatics, Redwood City, CA, USA; https://www.qiagenbioinformatics.com). Significance was evaluated using Fisher's exact test and Benjamini–Hochberg adjusted *P* values (adjusted *p* < 0.05). Normalized and non‐normalized array data are accessible through the Gene Expression Omnibus with accession number GSE121970.

### Statistical analysis

Data are expressed as described in the figure and table legends. Mann–Whitney *U*‐tests were performed for comparisons between groups. Kruskal–Wallis one‐way analysis of variance test followed by a Dunn's multiple comparison test was used for groups of three or more. Survival rates are depicted by Kaplan–Meier plots and were compared by log‐rank test. Clinical observation scores were compared by calculating areas under the curves for each mouse followed by a *t*‐test. A *P* value below 0.05 was considered statistically significant. All analyses were performed using GraphPad Prism 5 (GraphPad Inc, San Diego, CA, USA).

## Results

### Inhibition of PKK gene expression and protein production by PKK ASO treatment

**Figure 1:**
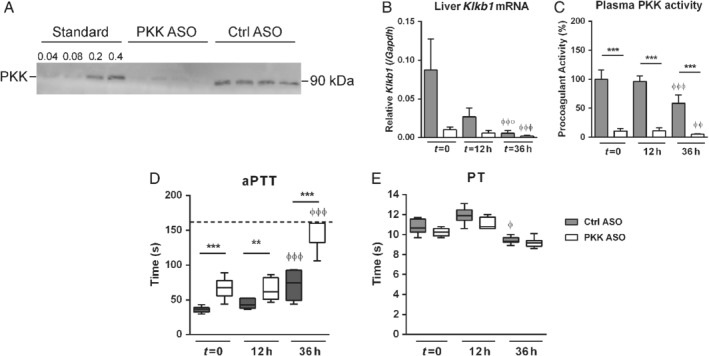
Inhibition of PKK expression by PKK ASO treatment. Mice were treated subcutaneously with PKK ASO (open bars) or control (Ctrl) ASO (grey bars) twice weekly for 3 weeks; 4 days after final dose, mice were infected with *K. pneumoniae* via the airways (*t* = 0). (A) Western blot of mouse plasma PKK (*t* = 0). Plasma (0.2 μl diluted in buffer; total volume 10 μl) from four PKK ASO‐ or four Ctrl ASO‐treated mice was loaded; ‘standard’ indicates volumes (μl) of plasma in buffer (total volume 10 μl) from one uninfected mouse (treated with Ctrl ASO). The blot shown is representative of three independent blots. (B) *Klkb1* mRNA expression in livers of mice treated with PKK or Ctrl ASO at corresponding time points. (C) Plasma PKK activity. (D) Activated partial thromboplastin time (aPTT). (E) Prothrombin time (PT). Data are mean and SD for bar charts in B and C or medians for box and whisker plots in D and E, eight mice per group. **p < 0.01, ***p < 0.001 (versus Ctrl ASO at the same time point); ^φ^ *p* < 0.05, ^φφ^ *p* < 0.01, ^φφφ^ *p* < 0.001 (versus *t* = 0 in the same treatment group).

In order to suppress expression of *Klkb1* (the gene encoding PKK), mice were treated twice weekly with a PKK ASO for 3 weeks according to a previously described dosing schedule 18, 19; control mice were treated with a scrambled Ctrl ASO. After 3 weeks of treatment (designated *t* = 0 in the infection experiments described below), *Klkb1* mRNA expression in the liver was inhibited by over 80% in PKK ASO‐treated mice compared with mice treated with Ctrl ASO (Figure 1B). Consistently, plasma PKK protein and activity were almost completely abolished in PKK ASO‐treated mice (Figure 1A,C). In accordance, PKK ASO administration was associated with a strong prolongation of the aPTT (Figure 1D), while the PT remained unaffected (Figure 1E). *Klkb1* mRNA was not detectable in lungs. Having established that PKK ASO treatment efficiently reduced PKK expression, we went on to infect PKK ASO‐ and Ctrl ASO‐injected mice with *Klebsiella* via the airways. The effect of PKK ASO administration on liver *Klkb1* mRNA, plasma PKK activity, and the aPTT was sustained for at least 36 h after infection (Figure 1A–D). Notably, in Ctrl ASO‐treated mice, liver *Klkb1* mRNA and plasma PKK activity levels decreased during the infection (Figure 1B,C), which was accompanied by a prolongation of the aPTT over time (Figure 1D). The activities of other components of the contact system did not decrease (FXII) or even increased (HK) during infection (supplementary material, Figure S1A,B). PKK ASO treatment was associated with elevated plasma FXII activity at *t* = 0 and *t* = 12 h, and with reduced plasma HK activity at 36 h. PKK ASO administration diminished activation of coagulation, as indicated by an attenuated rise in plasma TATc levels (supplementary material, Figure S1C).

### PKK depletion improves host defense during pneumosepsis

**Figure 2:**
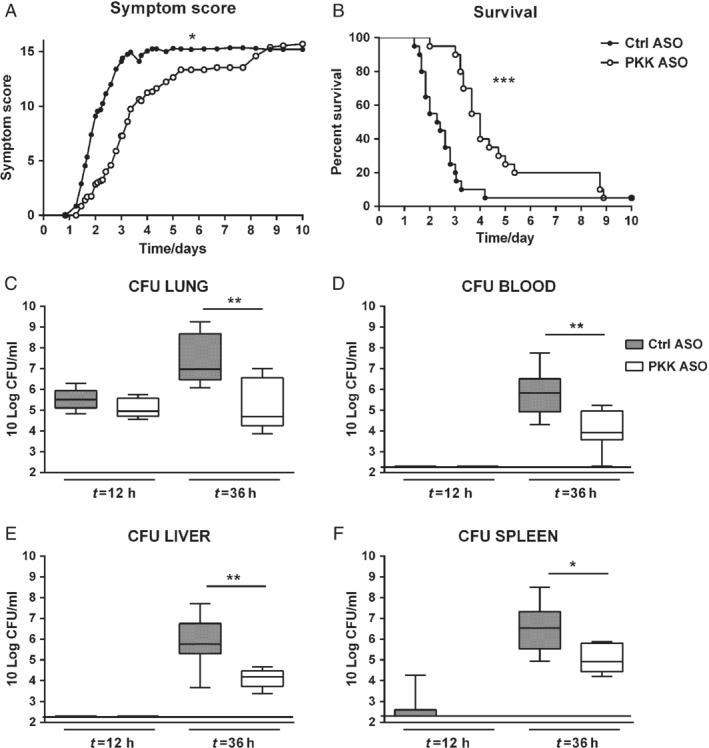
PKK depletion results in a prolonged survival associated with attenuated bacterial growth and dissemination during pneumosepsis. Mice were treated subcutaneously with PKK ASO (open dots and bars) or control (Ctrl) ASO (filled dots and bars) twice weekly for 3 weeks; 4 days after final dose, mice were infected with *K. pneumoniae* via airways (*t* = 0) and either followed for 10 days (A, B; *n* = 20 per group) or euthanized at 12 or 36 h after infection (C–F; *n* = 8 per group at each time point). (A) Symptom score (see the Materials and methods section for details). (B) Survival. (C–F) Bacterial loads in lungs, blood, liver, and spleen. Data are presented as box and whisker plots with medians. *p < 0.05, **p < 0.01, ***p < 0.001 (all versus Ctrl ASO).

To obtain a first insight into the role of PKK in host defense during pneumosepsis, we infected mice pretreated with either PKK or Ctrl ASO with *Klebsiella* via the airways and observed them for 10 days. Ctrl ASO mice started to show symptoms 1 day after induction of pneumonia (Figure 2A), and most of them died between 40 and 72 h after infection, with a median survival time of 55 h (Figure 2B). In contrast, mice pretreated with PKK ASO died mostly between 72 and 120 h after infection (median survival time 96 h; *p* < 0.0005 versus Ctrl ASO mice). In accordance with the delayed mortality in PKK ASO‐treated mice, these animals displayed a postponed onset of symptoms (*p* < 0.05 versus Ctrl ASO mice).

We assessed whether the protective effect of PKK deletion was associated with an altered pattern of bacterial growth and dissemination. To this end, we determined the bacterial loads at the primary site of infection (lungs) and distant organs (blood, spleen, and liver) at an early (12 h) and a late time point (36 h; i.e. before the first deaths occurred) after infection. Consistent with the symptom scores, at 12 h, bacterial loads in the lungs were similar in PKK‐ and Ctrl ASO‐treated mice (Figure 2C); at this early time point, the vast majority of mice did not show evidence of dissemination of the infection (Figure 2D–F). At 36 h after infection, however, lung bacterial loads were approximately 100‐fold lower in PKK ASO‐treated mice relative to Ctrl ASO‐treated animals (*p* < 0.01); similarly, bacterial loads in blood, spleen, and liver were also 10‐ to 100‐fold lower in PKK ASO‐treated mice (*p* < 0.01 to *p* < 0.05). These results suggest that PKK depletion prolongs survival by reducing bacterial growth and dissemination during Gram‐negative pneumonia‐derived sepsis.

### Effect of PKK depletion on lung pathology and inflammation during pneumosepsis

The extent of lung inflammation increased in both groups after infection, and no difference was observed between PKK‐ and Ctrl ASO‐treated mice (supplementary material, Figure S2). Likewise, PKK depletion did not impact fibrin deposition in lung tissue (supplementary material, Figure S3). Considering that influx of neutrophils into the respiratory tract is a hallmark feature of bacterial pneumonia 34 and that contact system activation products can enhance neutrophil recruitment 9, 35, we next quantified neutrophil numbers in lung tissue using immunohistopathology and digital imaging (supplementary material, Figure S4A,B). Pneumonia was associated with a gradual increase in neutrophils in lung tissue, which was not different between PKK‐ and Ctrl ASO‐treated mice. Consistently, neither total cell counts (supplementary material, Figure S4C) nor neutrophil numbers (supplementary material, Figure S4D) in the bronchoalveolar space differed between treatment groups.

To further study the effect of PKK depletion on lung inflammation after infection, we measured the levels of cytokines (TNFα, IL‐1β, IL‐6) and chemokines (CXCL1, CXCL2) in lung homogenates (Table 1). Directly prior to infection, PKK ASO‐treated mice tended to have higher lung levels of these pro‐inflammatory mediators when compared with Ctrl ASO‐injected mice (although not significantly higher). After infection, pulmonary cytokine and chemokine levels were lower in PKK ASO‐treated mice than in Ctrl ASO‐treated mice, albeit with a large inter‐individual variation; significant differences were found for IL‐6 and CXCL1 at 12 h after infection, and for IL‐6, IL‐1β, and CXCL2 at 36 h after infection.

**Table 1:** Effect of PKK depletion on lung cytokine and chemokine levels during Gram‐negative pneumosepsis

	*t* = 0		*t* = 12 h		*t* = 36 h	
Cytokine (pg/ml)	Ctrl ASO	PKK ASO	Ctrl ASO	PKK ASO	Ctrl ASO	PKK ASO
TNF‐α	1022 (712.9–1962)	1900 (1362–2341)	7358 (5092–11 300)	5665 (3290–6277)	10 589 (7539–17 907)	7333 (5318–13 312)
IL‐1β	173.8 (24.64–356.6)	232.7 (157.4–409.4)	874.1 (496.2–1197)	576.4 (401.1–760.8)	831.7 (582.9–1175)	537.8* (357.2–709.6)
IL‐6	274.3 (118.6–527)	430 (204.4–571.5)	3663 (1545–7194)	1460* (970–1733)	2280 (1371–3493)	1116* (905.7–1741)
CXCL1	292.3 (176.7–464.9)	449 (372.4–848.4)	6375 (3210–10 139)	2491* (2231–2987)	7122 (6032–7965)	5466 (4610–7223)
CXCL2	268.9 (156.4–577.1)	556.7 (412.4–704.2)	4880 (2954–8442)	2786 (2003–3217)	5418 (3802–7519)	1925* (1322–4152)

Lung cytokine and chemokine levels before and 12 and 36 h after induction of *Klebsiella* pneumonia in Ctrl ASO‐ and PKK ASO‐treated mice. Data are expressed as median (inter‐quartile ranges) of eight mice per group at each time point.

* *p* < 0.05 versus Ctrl ASO group, Mann–Whitney test.

### PKK depletion attenuates distant organ damage during pneumosepsis

**Figure 3:**
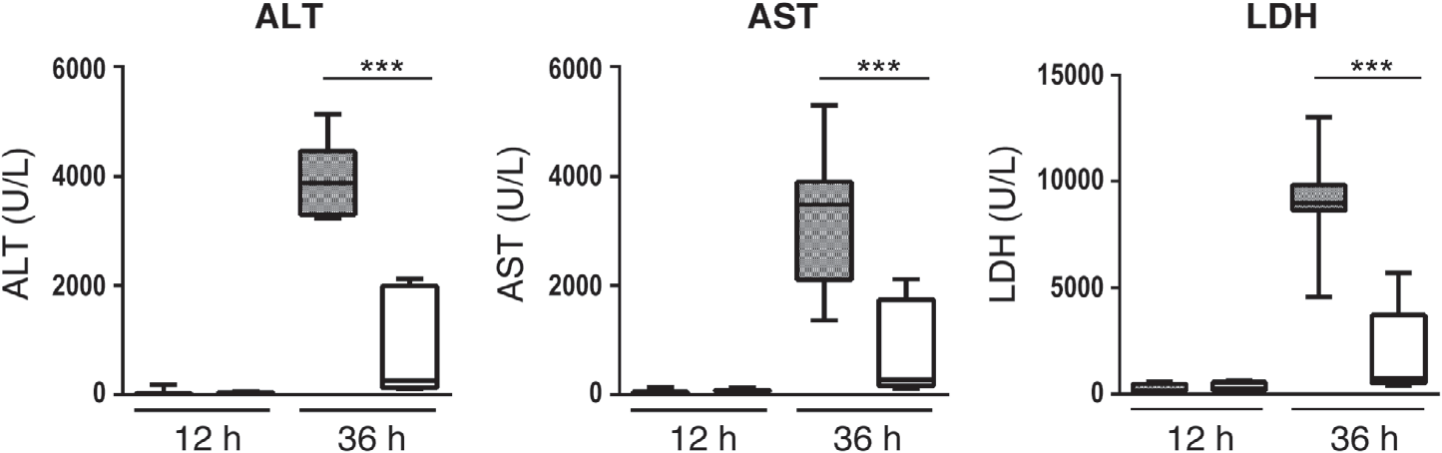
PKK depletion attenuates distant organ injury. Mice were treated subcutaneously with PKK ASO (open bars) or control ASO (grey bars) twice weekly for 3 weeks; 4 days after final dose, mice were infected with *K. pneumoniae* via airways (*t* = 0) and euthanized at 12 or 36 h after infection (*n* = 8 per group at each time point). Data are shown as box and whisker plots with medians. ***p < 0.001.

To obtain further insight into the mechanism by which PKK depletion affected outcome, we measured plasma ALT and AST as markers of hepatocellular injury and plasma LDH to indicate cellular injury in general. Plasma ALT, AST, and LDH levels increased markedly during the late stage of sepsis in both treatment groups, but significantly less so in PKK ASO‐treated mice (Figure 3). Moreover, Ctrl ASO‐ but not PKK ASO‐injected mice showed thrombus formation in liver tissue 36 h after infection (supplementary material, Figure S5).

### *Klebsiella* does not activate the contact system

**Figure 4:**
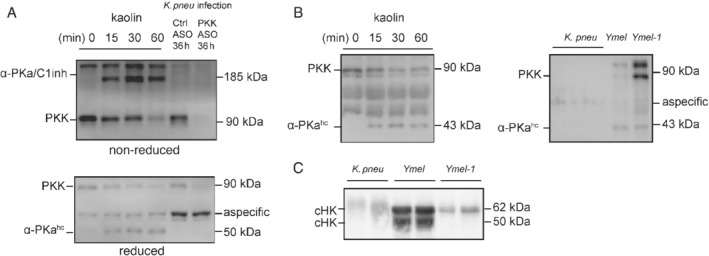
*Klebsiella pneumoniae* does not activate the contact system *in vivo* or *in vitro*. Mice were treated subcutaneously with PKK ASO (open bars) or control ASO (grey bars) twice weekly for 3 weeks; 4 days after final dose, mice were infected with *K. pneumoniae* via airways (*t* = 0) and euthanized at 12 or 36 h after infection. (A) Western blots showing full length PKK and the cleaved chain of α‐PKa in plasma obtained 36 h after infection using either reduced or non‐reduced samples; naive mouse plasmas pretreated with 100 μg/ml kaolin for the indicated time (15 min to 1 h) were used as positive controls. (B, C) *K. pneumoniae*, *E. coli* Ymel or *E. coli* Ymel‐1 was incubated in citrated plasma from healthy volunteers and bacterial bound PKK (B) and HK (C) proteins were detected by western blotting after reducing SDS‐PAGE. cHK, cleaved chains of HK; α‐PKa^hc^, α‐PKa heavy chain.

To study activation of the contact system and the role of PKK herein, we measured cleaved PK and HK by immunoblotting analysis of plasma from infected mice and human plasma exposed to *K. pneumoniae in vitro*. Kaolin (positive control) activated PKK to α‐PKa, which is immediately captured by C1 inhibitor in plasma, as detected by the appearance of α‐PKa/C1 inhibitor complexes (185 kDa product; non‐reduced condition) 36 and α‐PKa heavy chain generation (reduced condition; heavy chain is liberated of the serine protease domain containing light chain that is captured by C1 inhibitor) in mouse plasma (Figure 4A). In contrast, neither α‐PKa/C1 inhibitor nor α‐PKa heavy chain was detected in the plasma of Ctrl ASO‐ or PKK ASO‐treated mice infected with *Klebsiella* (Figure 4A). We further tested whether the *K. pneumoniae* strain used in our *in vivo* studies can activate the contact system *in vitro*, using *E. coli* Ymel (able to bind both PKK and HK) and the *E. coli* mutant Ymel‐1 (not able to bind PKK or HK) as comparators 20. Neither full length PKK nor α‐PKa heavy chain was detectable on *K. pneumoniae* incubated with plasma, indicating that PKK was not absorbed/activated on the cell surface of this bacterium (Figure 4B). PKK and activation products could be detected at the surface of *E. coli* Ymel and, to a limited extent, of the *E. coli* mutant Ymel‐1. Likewise, HK could be bound and cleaved at the surface of *E. coli* Ymel (cHK), while not so on the surface of *Klebsiella* or *E. coli* Ymel‐1 (Figure 4C).

### PKK depletion is associated with an upregulation of multiple innate immune pathways in the lungs

**Figure 5:**
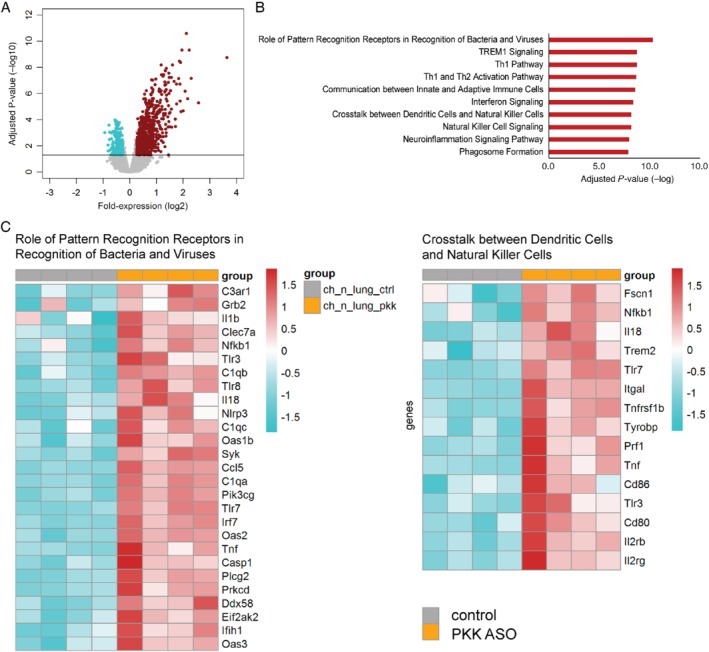
PKK depletion results in enhanced expression of multiple innate immune pathways in lungs of uninfected mice. Mice were treated subcutaneously with PKK ASO or control ASO twice weekly for 3 weeks and euthanized 4 days after final dosing. RNA was purified from whole lungs and genome‐wide mRNA expression was analyzed. (A) Volcano plots [integrating adjusted *P* values and fold‐expression (log_2_ fold‐change)] depicting the global alteration in gene expression after treatment with PKK ASO relative to control ASO administration. Horizontal line indicates Benjamini–Hochberg (BH) adjusted *p* < 0.05. Red dots denote overexpressed genes; turquoise dots indicate underexpressed genes. (B) Ingenuity pathway analysis of elevated transcripts (red bars) with Benjamini–Hochberg (BH) adjusted Fisher's *p* < 0.01 demarcating significance; pathway analysis did not reveal significantly altered pathways for underexpressed genes. (C) Heatmap representation of transcript expression (rows) grouped as Ingenuity canonical signaling pathways in PKK ASO‐ and control ASO‐treated animals. Red, high expression; turquoise, low expression.

**Figure 6:**
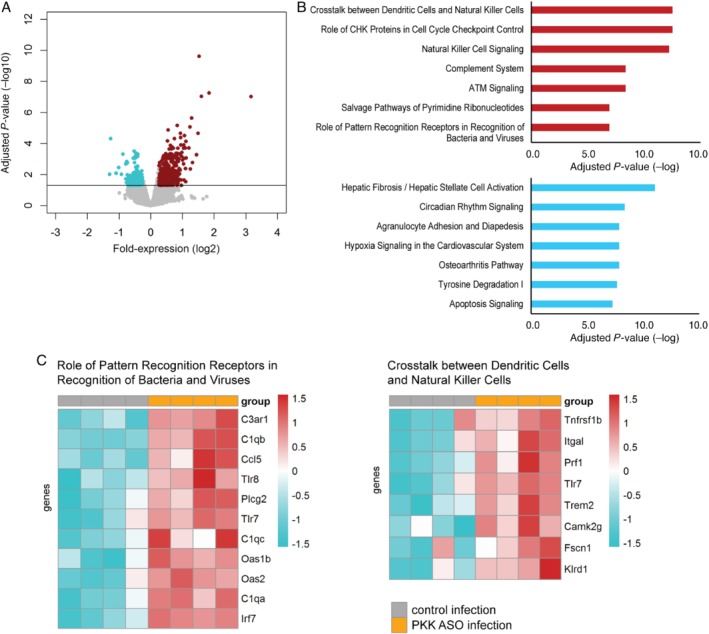
PKK depletion results in enhanced expression of multiple innate immune pathways in lungs of mice 12 h after infection. Mice were treated subcutaneously with PKK ASO or control ASO twice weekly for 3 weeks, infected with *K. pneumoniae* 4 days after final dosing, and euthanized 12 h later. RNA was purified from whole lungs and genome‐wide mRNA expression was analyzed. (A) Volcano plots [integrating adjusted *P* values and fold‐expression (log_2_ fold‐change)] depicting the global alteration in gene expression after treatment with PKK ASO relative to control ASO administration. Horizontal line indicates Benjamini–Hochberg (BH) adjusted *p* < 0.05. Red dots denote overexpressed genes; turquoise dots indicate underexpressed genes. (B) Ingenuity pathway analysis of elevated transcripts (red bars) and reduced transcripts (turquoise bars) with Benjamini–Hochberg (BH) adjusted Fisher's *p* < 0.01 demarcating significance. (C) Heatmap representation of transcript expression (rows) grouped as Ingenuity canonical signaling pathways in PKK ASO‐ and control ASO‐treated animals. Red, high expression; turquoise, low expression.

To gain insight into the mechanism by which PKK modulates the host response during pneumonia‐derived sepsis, we chose an unbiased approach and investigated the effect of PKK depletion on lung and liver transcriptomes. When comparing lung transcriptomes of uninfected PKK ASO‐treated mice with those of Ctrl ASO‐administered mice, 1237 significantly altered genes were revealed (Figure 5A). Pathway analysis revealed that overexpressed genes in PKK ASO‐treated mice were significantly associated with pathogen recognition and several innate/adaptive immunity pathways including TREM‐1 signaling, interferon signaling, Th1 pathway, and crosstalk between dendritic cells and natural killer cells (Figure 5B,C). Infection with *Klebsiella* caused a profound change in the lung transcriptome relative to uninfected mice characterized by enhanced expression of several innate immune pathways (supplementary material, Figure S6A,B). The overexpression of several pathways in naïve mice treated with PKK ASO was sustained at 12 h after infection, most notably so for pathogen recognition, crosstalk between dendritic cells and natural killer cells, and natural killer signaling, while at this time point also complement signaling was enhanced in PKK ASO‐injected mice (Figure 6A–C). Pathways that were underexpressed in PKK ASO‐treated mice, amongst others, related to fibrosis and agranulocyte adhesion and diapedesis. The array analyses at *t* = 0 and *t* = 12 h confirmed that *Klkb1* mRNA was not expressed in the lungs (data not shown). In the liver, transcriptome analysis also pointed to a marked activation of several innate and adaptive immune pathways in PKK‐depleted mice, both directly prior to and 12 h after infection (supplementary material, Figures S7A,B and S6C,D, respectively). By comparison, infection *per se* induced less pronounced upregulation of mRNAs in liver 12 h after infection (supplementary material, Figure S7E,F).

## Discussion

We have used an established model of lower respiratory tract infection caused by the common human pathogen *K. pneumoniae* to investigate the role of PKK in host defense. This model is characterized by infection with a low bacterial dose via the airways initially resulting in a localized infection contained within the lungs and subsequently in sepsis with distant organ injury and death, thereby allowing for studies on both the early protective immune response and the late detrimental consequences of ongoing immune activation 21, 22, 23. At the initiation of our investigations on the role of the kallikrein–kinin system in pneumonia‐derived sepsis, we anticipated that an impairment of the function of this pluripotent pro‐inflammatory system, such as by depletion of PKK, would impair the early innate immune response and result in enhanced bacterial growth. During later phases of the infection, PKK depletion in theory could have protected against organ injury, consistent with earlier studies that documented the harmful effects of activation products of the kallikrein–kinin system in models of acute fulminant infection or endotoxemia 12, 13. In contrast to these expectations, PKK depletion was associated with enhanced expression of multiple innate immune pathways in lungs and liver, together with an improved antibacterial defense. While hepatic PKK production and plasma PKK activity decreased during the progression of pneumonia‐derived sepsis in control animals, mice in which PKK was depleted prior to infection showed delayed mortality together with reduced distant organ injury prior to the occurrence of the first deaths, most likely due to limitation of bacterial spread. Considering that we could not detect *Klkb1* mRNA in lungs, these data suggest that circulating PKK, originating from constitutive PKK production in the liver, restrains pro‐inflammatory signaling in the lung, which in the case of respiratory tract infection may impede early protective innate immunity.

We recently reported on the role of other components of the contact system in the host response during pneumonia‐derived sepsis using the same model. Neither kininogen depletion (by ASO treatment) nor kininogen deficiency influenced bacterial growth or inflammatory responses after infection with *K. pneumoniae* via the airways 15. Likewise, neither bradykinin receptor deficiency nor inhibition modified the host response in this model 16. These earlier data and the fact that we were unable to detect activation of the contact system suggest that the mechanism by which PKK impacted host defense is unrelated to its role in the kallikrein–kinin system. While to the best of our knowledge zymogen PKK has not been linked with regulation of innate immunity, zymogen FXII has recently been shown to enhance several innate immune functions of neutrophils 37. Other findings have also indicated that zymogen FXII may influence cell functions independent of its enzymatic action 38. Notably, we previously documented that kininogen‐depleted or ‐deficient mice have much lower plasma PKK levels (∼40% of the PKK levels in the respective control mice) 15. We consider it likely that the markedly different phenotypes of kininogen‐depleted/deficient mice versus PKK‐depleted mice during *Klebsiella* pneumosepsis relate to the different extents of plasma PKK reduction, which was virtually complete in PKK ASO‐treated mice.

PKK ASO treatment resulted in a sustained inhibition of *Klkb1* mRNA expression in the liver and a profound reduction in plasma PKK activity. This effect was associated with induction of multiple innate immune pathways already prior to infection, especially in the lung. These immune‐enhancing effects were not associated with changes in lung pathology or recruitment of white blood cells or neutrophils to the bronchoalveolar space (supplementary material, Figure S4), or of neutrophils (supplementary material, Figure S4), lymphocytes or macrophages (data not shown) to lung tissue, suggesting that these remained restricted to an extent not causing autoinflammation. Nonetheless, these pro‐inflammatory modifications of innate immune signaling pathways are a likely explanation for the observed protective effect of PKK ASO treatment. Indeed, in general, early stimulation of innate immunity in the lung is associated with an improved defense against respiratory pathogens in pneumonia models 34, and specific pathways induced by PKK ASO administration such as pattern recognition receptors 39, 40, TREM‐1 signaling 41 and interferon signaling 42, 43 have been implicated in protective immunity during *Klebsiella* pneumonia (supplementary material, Figure S8). A non‐specific pro‐inflammatory effect of PKK ASOs is unlikely considering that scrambled Ctrl ASOs were used in all experiments. In addition, unlike the strong improvement of host defense provided by PKK ASO treatment, ASOs targeting kininogen 15 or FXII 44 did not influence bacterial loads or inflammatory responses in the same model of *Klebsiella*‐induced pneumosepsis.

Progressive infection was accompanied by a selective decline in plasma PKK activity; plasma FXII and plasma HK activity remained unaltered or increased, respectively. It is tempting to speculate that this decline in systemic PKK levels serves to facilitate the innate immune response in the lungs during pneumonia. PKK depletion was associated with elevated plasma FXII activity, which is in accordance with a previous study 18 and consistent with FXII being a substrate for activated PKK.

PKK depletion was associated with reduced distant organ injury. Although earlier investigations documented an injurious role for contact system activation in fulminant models of endotoxemia and sepsis 12, 13 in the present investigation activation of the contact system could not be detected and the protective effect of PKK ASO administration likely was due to the lower bacterial burdens. Likewise, while previous studies have documented a role for PKK in thrombosis 18, 45, diminished thrombus formation in livers of PKK ASO‐treated mice could also be explained by the lower bacterial burdens in these animals, providing a reduced pro‐coagulant stimulus. This would also explain the lower plasma TATc levels in PKK ASO‐administered animals during late‐stage infection. Alternatively, and not mutually exclusive, severe infection such as induced here is likely to result in significant hypoxia, which could explain differences in hepatocellular injury between groups, considering the less severe disease course in PKK‐depleted mice.

Our study is limited by the fact that we do not provide a mechanism by which PKK influences innate immune pathways. In addition, gene expression profiles were studied in tissues and the effect of PKK depletion on specific cell types remains to be established. Moreover, we did not study the effect of PKK or PKK‐depleted serum on the killing capacity of phagocytes; for the results presented here, this would have been difficult considering that the highly virulent *Klebsiella* strain used cannot be killed by phagocytes *in vitro* 46. Additional experiments using purified PKK and purified cells are warranted to obtain more insight into the role of PKK in host defense and innate immune responses.

In conclusion, we have shown that constitutive production of PKK in the liver is diminished during pneumonia‐derived sepsis caused by the common human pathogen *K. pneumoniae*, resulting in declining plasma PKK concentrations in the absence of detectable contact system activation. PKK depletion prior to infection resulted in activation of multiple innate immune pathways in the lungs, which upon inoculation of *Klebsiella* via the airways was associated with reduced bacterial growth and dissemination, attenuated distant organ injury, and an improved survival. These results point to a novel inflammation‐confining role of circulating PKK, which during a gradually evolving infection can impede protective immunity.

## Author contributions statement

CD, IS, and JY carried out experiments. BS analyzed array data. JR, OB, and AV analyzed pathology. PN contributed to array analyses. AR and JC generated antisense oligonucleotides and conceived experiments. CV and TP supervised the study. CD and TP drafted the manuscript. All the authors were involved in writing the paper and approved the final version.

## Supporting information


**Figure S1.** Effect of PKK ASO treatment on plasma FXII and HK activity and on activation of the coagulation system
**Figure S2.** PKK depletion does not influence lung pathology
**Figure S3.** PKK depletion does not impact fibrin(ogen) deposition in the lungs
**Figure S4.** PKK depletion does not influence leukocyte recruitment to lungs
**Figure S5.** PKK depletion prevents thrombus formation in the liver
**Figure S6.** Gene expression in lungs induced by *Klebsiella* pneumonia
**Figure S7.** PKK depletion results in enhanced expression of multiple pro‐inflammatory pathways in livers of uninfected mice
**Figure S8.** Schematic presentation of the effect of PKK depletion during *Klebsiella*‐induced pneumosepsis
