# Supplementary material for: Prekallikrein inhibits innate immune signaling in the lung and impairs host defense during pneumosepsis in mice
Source: J Pathol. 2019 Nov 25;250(1):95–106. doi: 10.1002/path.5354 (PMC6972537; doi:10.1002/path.5354)
Supplement: Supplementary file 1 — Figure S1. Effect of PKK ASO treatment on plasma FXII and HK activity and on activation of the coagulation system Figure S2. PKK depletion does not influence lung pathology Figure S3. PKK depletion does not impact fibrin(ogen) deposition in the lungs Figure S4. PKK depletion does not influence leukocyte recruitment to lungs Figure S5. PKK depletion prevents thrombus formation in the liver Figure S6. Gene expression in lungs induced by Klebsiella pneumonia Figure S7. PKK depletion results in enhanced expression of multiple pro‐inflammatory pathways in livers of uninfected mice Figure S8. Schematic presentation of the effect of PKK depletion during Klebsiella‐induced pneumosepsis [file PATH-250-95-s001.docx]

**Prekallikrein inhibits innate immune signaling in the lung and impairs host defense during pneumosepsis in mice**

Ding *et al. J Pathol* DOI: 10.1002/path.5354

**SUPPLEMENTARY FIGURES S1–S8**

**

**

**Figure S1 Effect of PKK ASO treatment on plasma FXII and HK activity and on activation of the coagulation system.** Mice were treated subcutaneously with PKK ASO (open bars) or control ASO (grey bars) twice weekly for 3 weeks; 4 days after final dose, mice were infected with *K. pneumoniae* via the airways (*t* = 0). (A) Plasma FXII activity. (B) Plasma HK activity. (C) Plasma TATc levels. Data are box and whisker plots with medians, eight mice per group. **p* < 0.05, ***p* < 0.01, ****p* < 0.001 versus control ASO; ^φφφ^*p* < 0.001 versus *t* = 0 in the same treatment group.


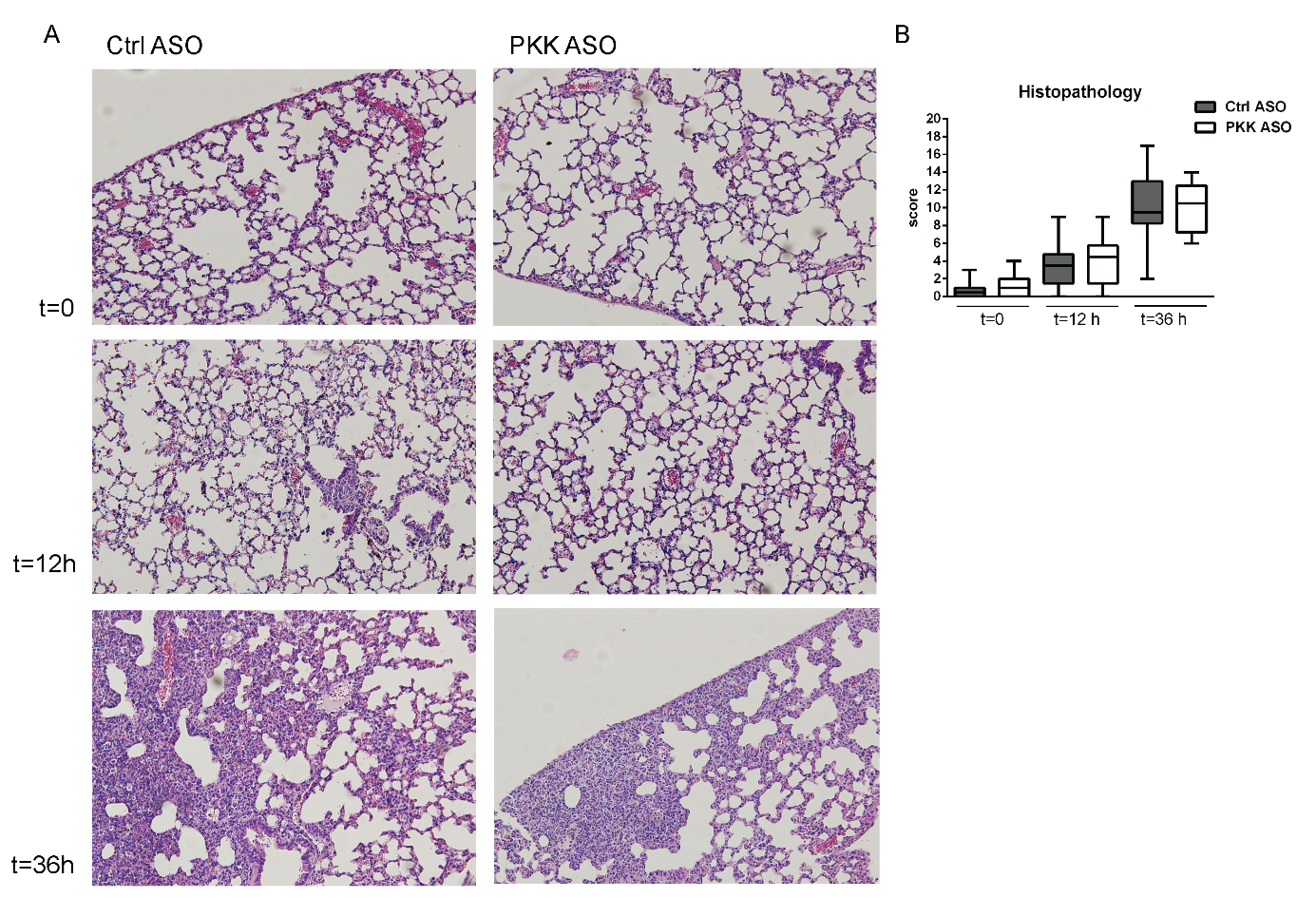


**Figure S2. PKK depletion does not influence lung pathology**.

Mice were treated subcutaneously with PKK ASO (open bars) or control ASO (grey bars) twice weekly for 3 weeks; 4 days after final dosing, mice were infected with *K. pneumoniae* via airways (*t* = 0) and euthanized at 12 or 36 h after infection (*n* = 8 per group at each time point). (A) Representative photographs of H&E-stained lung tissue sections (original magnification 4×). (B) The extent of inflammation scored on H&E tissue sections depicted as box and whisker plots with medians.

**
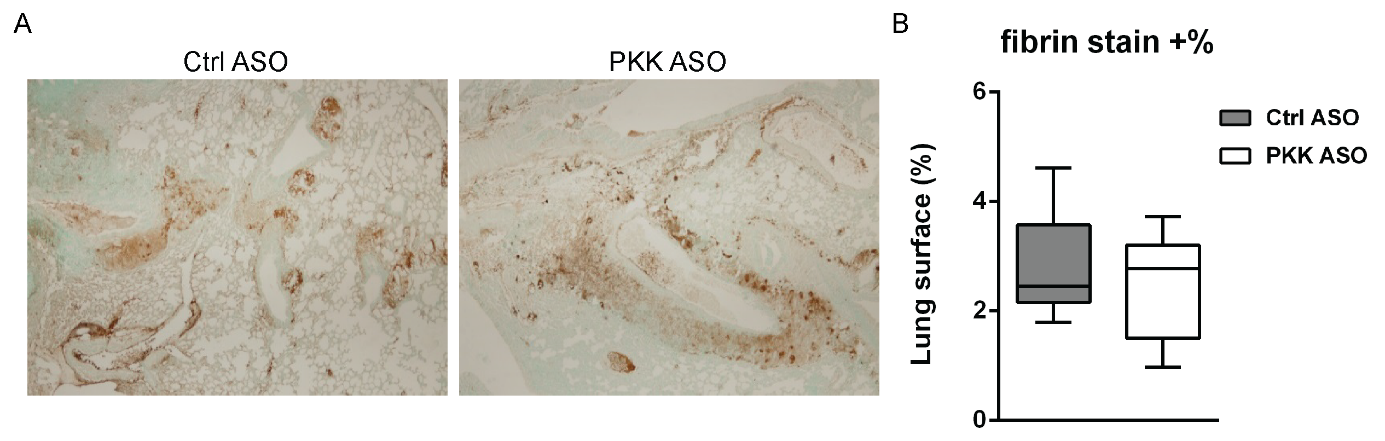
**

**Figure S3. PKK depletion does not impact fibrin(ogen) deposition in the lungs.**

Mice were treated subcutaneously with PKK ASO (open bars) or control ASO (grey bars) twice weekly for 3 weeks; 4 days after final dosing, mice were infected with *K. pneumoniae* via airways (*t* = 0) and euthanized at 36 h after infection (*n* = 8 per group). (A) Representative photographs of lung tissue sections stained for fibrin(ogen) (original objective magnification 4×). (B) The extent of positive staining as determined by imaging analysis depicted as box and whisker plots with medians. Differences between groups were not significant.

**
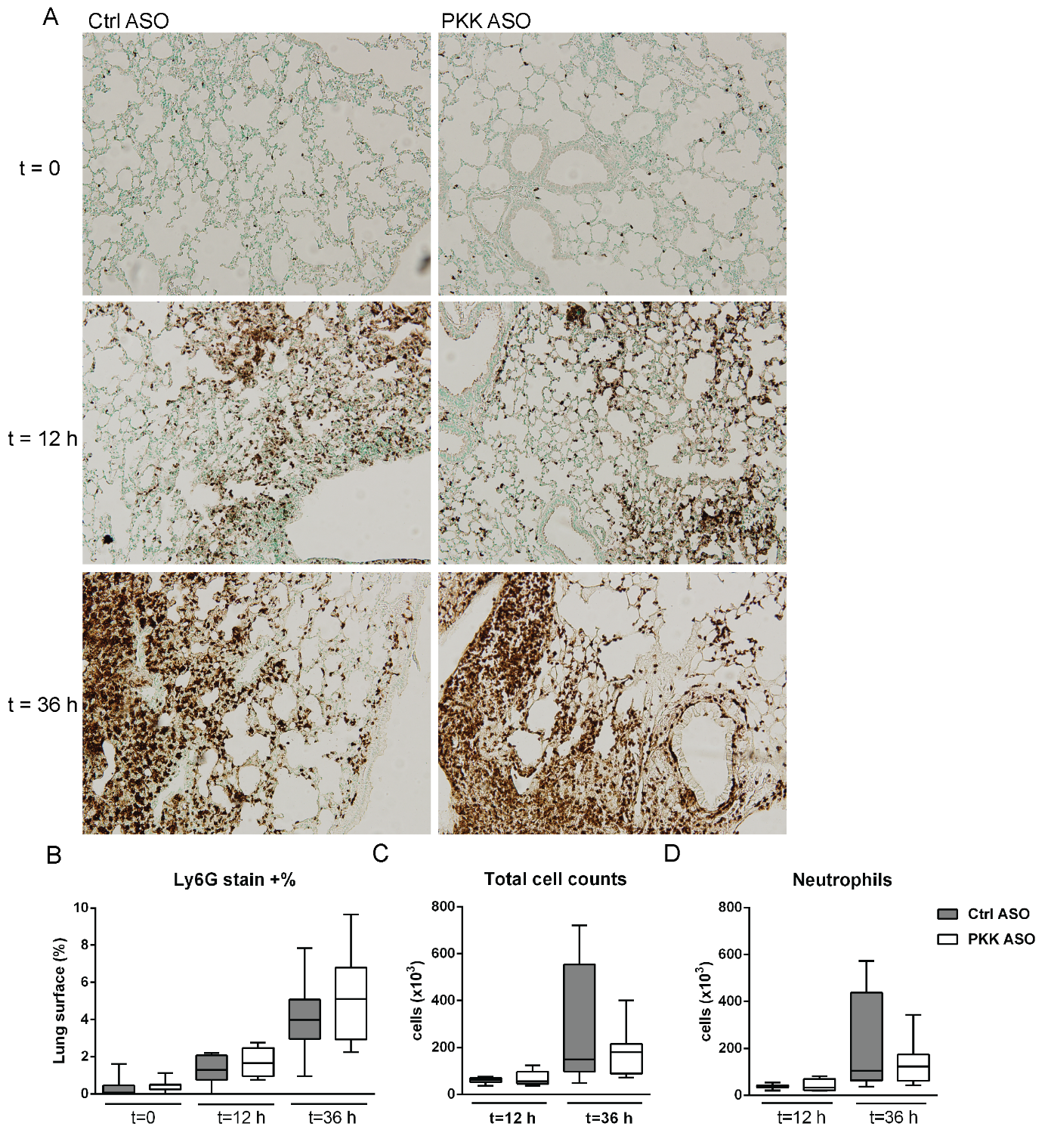
**

**Figure S4. PKK depletion does not influence leukocyte recruitment to lungs.**

Mice were treated subcutaneously with PKK ASO (open bars) or control ASO (grey bars) twice weekly for 3 weeks; 4 days after final dosing, mice were infected with *K. pneumoniae* via airways (*t* = 0) and euthanized at 12 or 36 h after infection (*n* = 8 per group at each time point). (A) Representative images of Ly-6G-stained tissue sections of non-infected (*t* = 0) and infected lungs (*t* = 12 and 36 h) (4× original magnification). (B) Quantification of the positive areas of immunohistochemistry staining for Ly-6G in the lung by ImageJ. (C, D) Total cell counts and absolute neutrophil numbers in bronchoalveolar lavage fluid. Panels B–D show box and whisker plots with medians. Differences between groups were not significant.


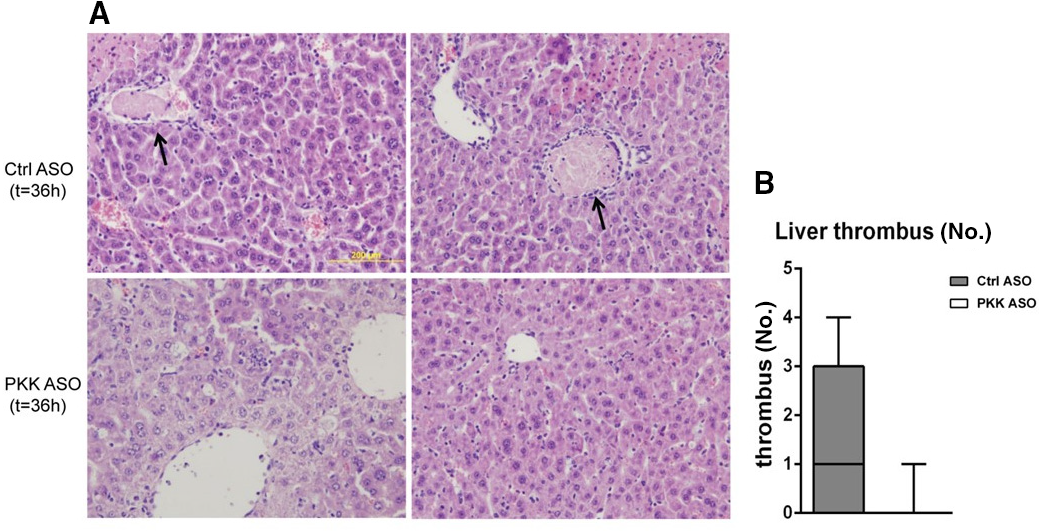


**Figure S5. PKK depletion prevents thrombus formation in the liver.**

Mice were treated subcutaneously with PKK ASO (open bars) or control ASO (grey bars) twice weekly for 3 weeks; 4 days after final dosing, mice were infected with *K. pneumoniae* via airways (*t* = 0) and euthanized at 12 or 36 h after infection (*n* = 8 per group at each time point). (A) Representative photographs of H&E-stained liver tissue sections (original magnification 4×) 36 h after infection (arrows indicate thrombi). (B) The extent of thrombus formation depicted as box and whisker plots with medians.

**
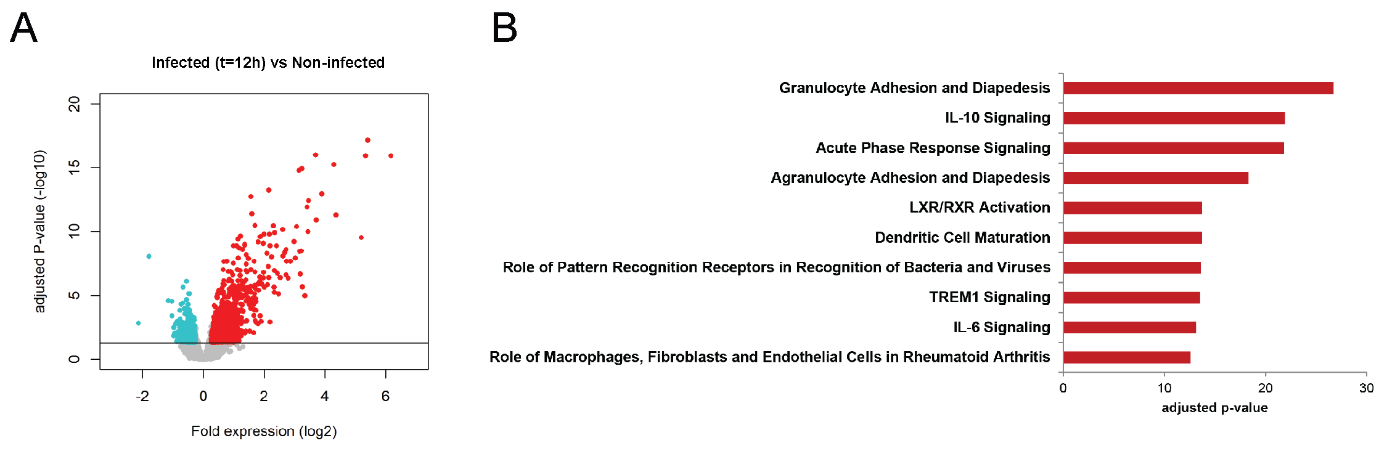
 Figure S6. Gene expression in lungs induced by *Klebsiella* pneumonia**.

Mice were treated subcutaneously with control ASO twice weekly for 3 weeks and infected with *K. pneumoniae* via the airways 4 days after final dosing. RNA was purified from whole lungs obtained directly before and at 12 h after infection, and genome-wide mRNA expression was analyzed. (A) Volcano plots [integrating adjusted *P* values and fold-expression (log_2_)] depicting the global alteration in gene expression after infection. Horizontal line indicates Benjamini–Hochberg (BH) adjusted *p* < 0.05. Red dots denote overexpressed genes; turquoise dots indicate underexpressed genes. (B) Ingenuity pathway analysis of elevated transcripts (red bars) with Benjamini–Hochberg (BH) adjusted Fisher’s *p* < 0.01 demarcating significance; pathway analysis did not reveal significantly altered pathways for underexpressed genes.


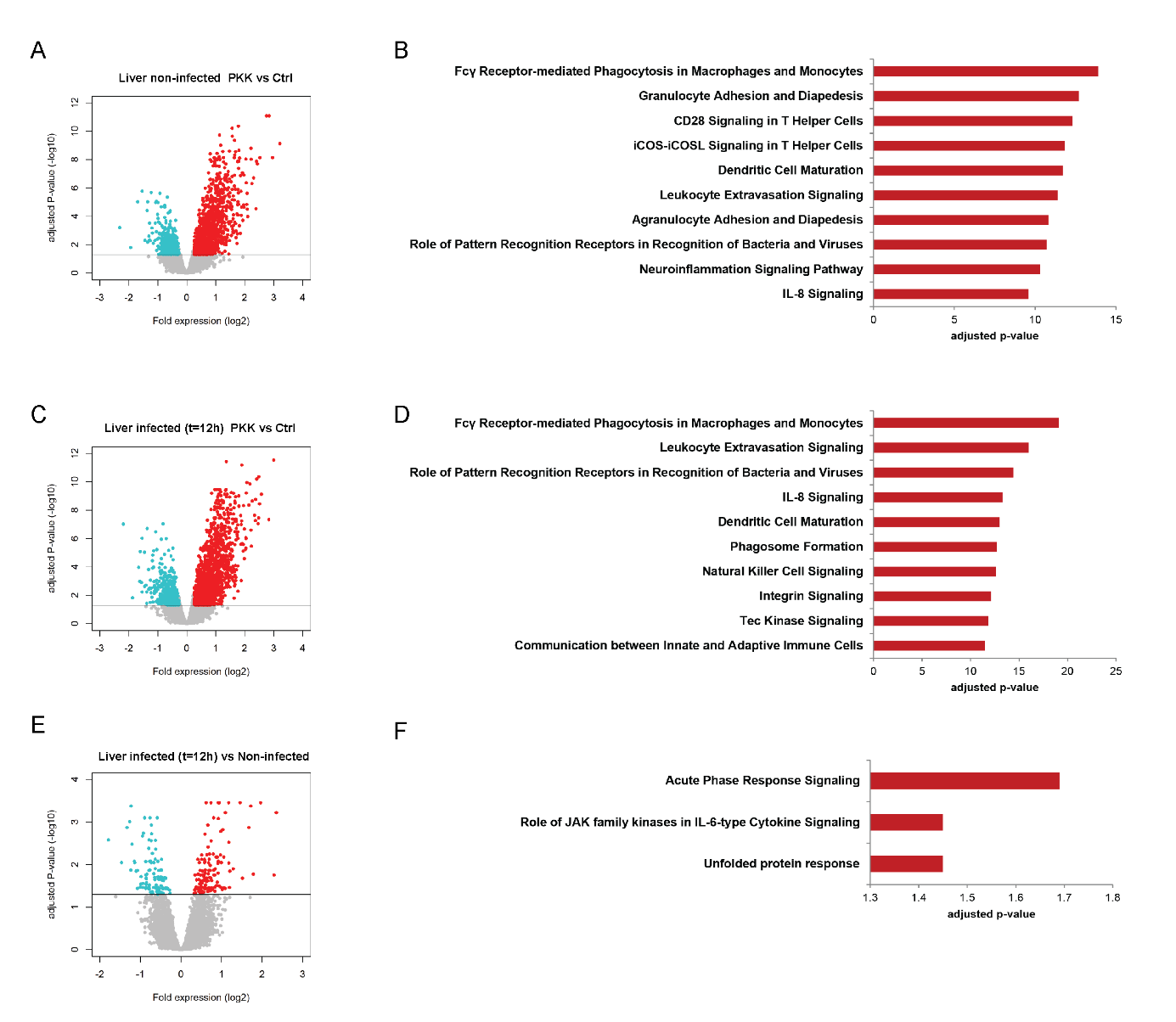


**Figure S7. PKK depletion results in enhanced expression of multiple pro-inflammatory pathways in livers of uninfected mice.**

Mice were treated subcutaneously with PKK ASO or control ASO twice weekly for 3 weeks and euthanized 4 days after final dosing (A) or 12 h after infection (B, C). RNA was purified from liver and genome-wide mRNA expression was analyzed. (A, C, E) Volcano plots [integrating adjusted *P* values and fold-expression (log2)] depicting the global alteration in gene expression after treatment with PKK ASO relative to control ASO administration before infection (A) or 12 h after infection (C); panel E compares infected (*t* = 12 h) with uninfected mice (*t* = 0) treated with control ASO. Horizontal line indicates Benjamini–Hochberg (BH) adjusted *p* < 0.05. Red dots denote overexpressed genes; turquoise dots indicate underexpressed genes. (B, D, F) Ingenuity pathway analysis of elevated transcripts in panels A, C, and E, respectively (red bars), with Benjamini–Hochberg (BH) adjusted Fisher’s *p* < 0.01 demarcating significance; pathway analysis did not reveal significantly altered pathways for underexpressed genes.


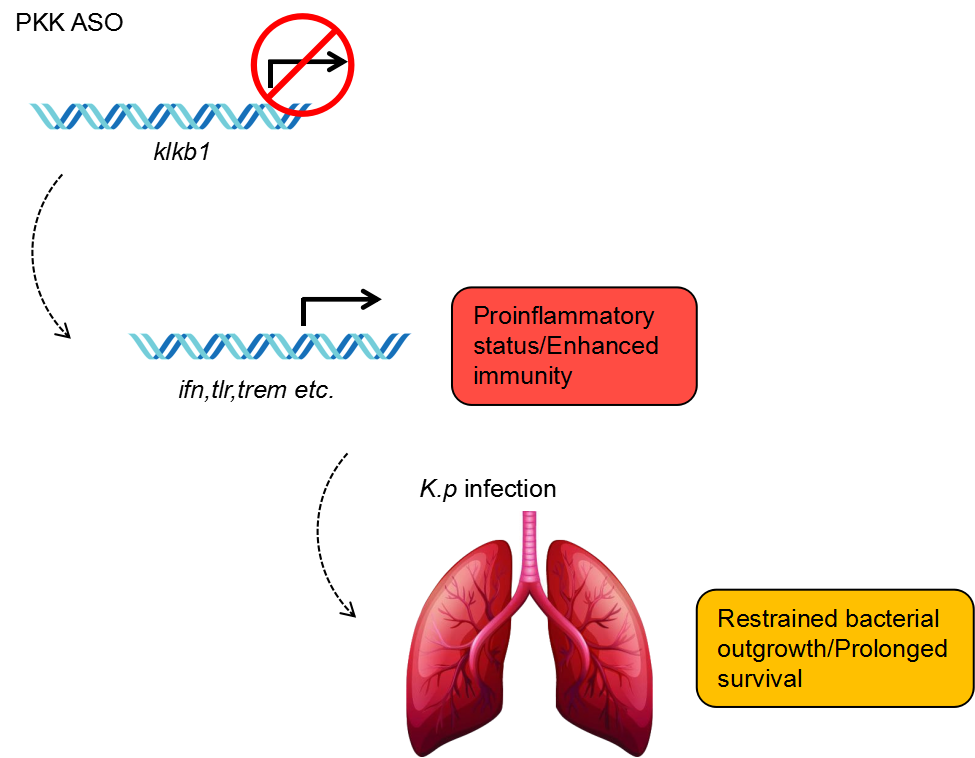


**Figure S8. Schematic presentation of the effect of PKK depletion during *Klebsiella*-induced pneumosepsis.**
